# Protective role of berberine in isoprenaline-induced cardiac fibrosis in rats

**DOI:** 10.1186/s12872-019-1198-9

**Published:** 2019-10-15

**Authors:** Yan Che, Di-Fei Shen, Zhao-Peng Wang, Ya-Ge Jin, Qing-Qing Wu, Sha-Sha Wang, Yuan Yuan

**Affiliations:** 1 Department of Cardiology, Renmin Hospital of Wuhan University, Jiefang Rd 238, Wuhan, 430060 China; 2 Hubei Key Laboratory of Metabolic and Chronic Diseases, Wuhan, China

**Keywords:** Berberine, Cardiac fibrosis, Macrophage, Fibroblast, TGF-β1, Cytokines

## Abstract

**Background:**

Cardiac fibrosis is a crucial aspect of cardiac remodeling that can severely affect cardiac function. Cardiac fibroblasts surely influence this process. Besides, macrophage plays an essential role in cardiac remodeling after heart injury. However, whether macrophage influence fibroblasts remain a question worth exploring. This study aimed to define the role of berberine (BBR) on isoprenaline (ISO)-induced cardiac fibrosis in an in vivo rat model and try to figure out the mechanism in vitro study.

**Methods:**

The Sprague-Dawley rats were divided into five groups: control group, ISO-treated group, and ISO + BBR (10 mg/kg/d, 30 mg/kg/d, and 60 mg/kg/d orally)-pretreatment groups. Fibrosis was induced by ISO administration (5 mg/kg/d subcutaneously) for 10 days. One day after the last injection, all of the rats were sacrificed. Using picrosirius red (PSR) straining, immunohistochemistry, immunofluorescence, flow cytometry, western blot, RT-qPCR and cell co-culture, we explored the influence of pretreatment by BBR on ISO-induced cardiac fibrosis.

**Results:**

Our results showed that BBR pretreatment greatly limited ISO-induced cardiac fibrosis and dysfunction. Moreover, BBR administration reduced macrophage infiltration into the myocardium of ISO-treated rats and inhibited transforming growth factor (TGF)-β1/smads signaling pathways in comparison to that seen in the ISO group. Besides, in vitro study showed that BBR-pretreatment reduced ISO-induced TGF-β1 mRNA expression in macrophages and ISO stimulation of macrophages significantly increased the expression of fibrotic markers in fibroblasts, but BBR-pretreatment blocked this increase.

**Conclusion:**

Our results showed that BBR may have a protective role to cardiac injury via reducing of macrophage infiltration and forbidding fibroblasts transdifferent into an ‘activated’ secretory phenotype, myofibroblasts.

## Background

Cardiac fibrosis is a requisite part of cardiac remodeling. The development of new therapies targeting cardiac fibrosis may limit cardiac remodeling and the subsequent development of heart failure. The activation of cardiac fibroblast transdifferentiation and the subsequent extracellular matrix deposition are key cellular events that drive the fibrotic response in the course of cardiac stress. It is worth noting that transforming growth factor (TGF)-β-producing inflammatory cells play a crucial role in this process [1]. It has been previously demonstrated that macrophages exert a wide range of actions that alter the extracellular matrix through phagocytosis and by the production of cytokines [tumor necrosis factor (TNF) α, interleukin (IL)-1β and IL-6], chemokines (monocyte chemotactic protein-1), and growth factors including TGF-β. Additionally, macrophages are always found in close proximity to collagen-producing myofibroblasts. Generally speaking, the pharmacological targeting of macrophages may provide effective therapies to prevent or inhibit cardiac fibrosis.

Chronic β-adrenergic stimulation using isoprenaline (ISO), a non-selective β-adrenergic receptor agonist, is sufficient to induce a myocardial proinflammatory response and myocardial fibrosis [2]. The administration of ISO to induce myocardial injury in Sprague-Dawley (SD) rats in the experimental setting has been commonly used in previous studies as to simulate β-adrenergic stimulation under stress conditions of heart [3–5]. In this study, we conducted myocardial injury models of SD rats using ISO administration same as previous studies.

Berberine (BBR), a bioactive alkaloid isolated from several herbal substances, possesses multiple pharmacological effects, including antimicrobial, antidiabetic, anticancer [6], anti-inflammatory, anti-oxidative, and cardioprotective properties [7]. Allijn et al. reported that BBR inhibited IL-6 secretion in macrophages and protected cardiac function against adverse remodeling for 28 days after a myocardial infarction [8]. In another study using an ISO-induced acute myocardial ischemia model in rats, BBR decreased serum levels of creatine kinase-MB, lactate dehydrogenase, TNF-α, and IL-6 through a regulation of the high mobility group box toll-like receptor 4 (HMGB1-TLR4) axis [9]. In the present study, we aimed to assess the protective effects of BBR administration in the prevention of ISO-induced cardiac fibrosis in rats and to study the underlying mechanisms associated with macrophages.

## Methods

### Reagents and animals

BBR (purity ≥98%) and ISO (purity ≥98%) were purchased from Sigma-Aldrich (Saint Louis, MO, USA). SD rats (male, 200–240 g) were purchased from the Beijing Vital River Laboratory Animal Technology Co., Ltd. (Beijing, China) and were kept under specific pathogen free conditions of housing and a 12-h light-dark cycle with free access to food and sterile water in the Cardiovascular Research Institute of Wuhan University (Wuhan, China) throughout the study. SD rats were randomly assigned to five groups of 15 rats each: (1) control; (2) ISO; (3) ISO + BBR (BBR 10 mg/kg/d, orally); (4) ISO + BBR (BBR 30 mg/kg/d, orally); and (5) ISO + BBR (BBR 60 mg/kg/d, orally). BBR doses used in the in vivo study of rat varies a lot from 5 mg/kg/d to 200 mg/kg/d in the cardiovascular models [10–12]. In the ISO-induced heart injury model, there has not been one reference concentration, so we choose three concentrations including 10 mg/kg/d, 30 mg/kg/d, and 60 mg/kg/d to explore the effect of BBR on the ISO-induced rat hearts. Rats were pretreated for 14 days with BBR (dissolved in sterile water) and were then treated with ISO (5 mg/kg/d with the exception of the control group, dissolved in sterile 0.9% saline) by subcutaneous injection for 10 consecutive days [13]. On the 11th day, the rats were anesthetized with 1.5% isoflurane and subjected to echocardiography and hemodynamic analysis. Subsequently, the rats were sacrificed by cervical dislocation while anesthetized.

### Echocardiography and catheter-based measurements of hemodynamic parameters

Left ventricular function was evaluated by echocardiography and catheter-based measurements of hemodynamic parameters. Briefly, after each rat was anaesthetized with chloral hydrate 10%, echocardiography was carried out using a Mylab 30CV (ESAOTE SpA; Florence, Italy) equipped with a 10-MHz linear array ultrasound transducer. Left ventricle (LV) dimensions were averaged from more than five cardiac cycles assessed in the parasternal short-axis view during systole or diastole. Interventricular septum thickness at diastole (IVSd) and left ventricular posterior wall thickness (LVPWd) were measured from the M-mode tracing with a sweep speed of 50 mm/s at the mid-papillary muscle level.

For hemodynamic measurements, after the induction of anesthesia with 1.5% isoflurane, a microtip catheter transducer (SPR-839, Millar Instruments; Houston, TX, USA) was inserted into the left ventricle of the rat via the right carotid artery. The signals were recorded using a Millar Pressure-Volume System (MPVS-400, Millar Instruments), and the end-diastolic pressure (EDP), end-systolic volume (ESV), time constant of isovolumic pressure decay (Tau_w), stroke volume (SV), ejection fraction (EF), and cardiac output (CO) were analyzed using PVAN data analysis software (Millar Instruments).

### Histological analysis

The animals were sacrificed after echocardiography and the catheter-based measurement of hemodynamic parameters. The hearts were removed from the rats, arrested in diastole with KCl 10%, and weighed after being wiped dry. The heart specimens were fixed with formaldehyde 4% before being embedded in paraffin. The rat hearts were cut transversely close to the apex to visualize the left and right ventricles. Thin tissue sections (4–5 μm thickness) were stained with picrosirius red (PSR) for histological analysis. Tissue sections were visualized by light microscopy.

### Quantitative real-time reverse transcription polymerase chain reaction

To examine the relative mRNA expression of CollagenIα, Collagen IIIα, connective tissue growth factor (CTGF), TGF-β1, and α-smooth muscle actin (SMA), total RNA was collected using TRIzol reagent (Invitrogen, Carlsbad, CA, USA) and the cDNA was used as a template for reverse transcription polymerase chain reaction (RT-PCR) amplification and detection of the gene expression level. Quantification RT-PCR was carried out using a one-step qPCR kit (Roche; Basel, Switzerland). PCR amplifications were quantified using a LightCycler 480 SYBR Green 1 Master Mix (Roche). The housekeeping gene, glyceraldehyde-3-phosphate dehydrogenase (GAPDH), was used to normalize gene mRNA expression.

### Western blotting

After homogenizing the tissues and cells using lysis buffer and centrifugation at 12,000 g for 20 min at 4 °C, protein amounts from all samples were measured with the BCA-kit (Thermo Fisher Scientific; Waltham, MA, USA). Protein samples (50 μg) were loaded onto sodium dodecyl sulfate polyacrylamide gel electrophoresis, and then transferred onto an immobilon-FL transfer membrane (Millipore, Billerica, MA, USA) in a transferring buffer. The membrane was blocked with 5% milk in tris-buffered saline tween-20 (TBST) for 1 h and then incubated overnight at 4 °C with antibodies against TGF-β1, p-smad2, smad2, p-smad3, smad3, smad4, and C-C chemokine receptor (CCR) 2, which were purchased from Cell Signaling Technology (Boston, MA, USA). GAPDH (MB001) was purchased from Bioworld Technology (St Louis Park, MN, USA). The blots were scanned using a two-color infrared imaging system (LI-COR Biosciences: Lincoln, NE, USA). Specific protein expression levels were normalized to GAPDH protein for total cell lysates.

### Immunohistochemistry

To visualize localization of CD68 in tissue sections, sections were stained with antibodies to CD45 (Abcam, ab10558) and CD68 (Abcam, ab955) for the identification of macrophages and 4′, 6-Diamidine-2′-phenylindole dihydrochloride (DAPI) for visualizing nuclei. Briefly, LV tissue sections were deparaffinized in xylene and dehydrated in a gradient concentration of ethanol. Antigen retrieval was performed by heat retrieval with citrate buffer for 20 min. The tissue slides were washed twice (5 min/wash) with tris-buffered saline (TBS) plus 0.025% Triton X-100 with gentle agitation and then blocked in 10% normal goat serum in TBS with 1% bovine serum albumin (BSA) for 2 h at room temperature. The primary antibody was diluted in TBS with 1% BSA. The specimens were incubated overnight at 4 °C and then rinsed twice (5 min/wash) in TBS plus 0.025% Triton X-100 with gentle agitation. Then the sections were incubated with EnVision™+/HRP reagent and stained with a DAB detection kit.

### Peritoneal macrophage culture

Primary peritoneal macrophages (2 × 10^6^ cells/well) of SD rats were cultured in 6-well plates (Corning; Corning, NY, USA) with 1 mL Roswell Park Memorial Institute medium (RPMI) supplemented with 20% fetal bovine serum (FBS) (HyClone; Logan, UT, USA) and allowed to attach for 1 h at 37 °C in 5% CO_2_. The adherent macrophages were cultured for an additional 24 h. For in vitro studies, ISO (20 mM) was dissolved in sterile 0.9% saline. BBR (20 mM) was dissolved in dimethyl sulfoxide (DMSO). To investigate TGF-β1 production in macrophages, different doses of BBR (0.1 μM, 0.5 μM, and 1 μM) were incubated with ISO (20 μM)-induced macrophages for 24 h. In order to investigate the effect of ISO on the macrophages, researchers used several concentrations of ISO, including 10 μM [14] and 50 μM [15]. In LPS-stimulated macrophages, researchers have applied several concentrations of BBR including 0.75 μM, 1.5 μM, 3 μM [16] and 20 μM [17]. In our research, at first, we designed 5 concentrations (0.1 μM, 0.5 μM, 1 μM, 5 μM, 20 μM), under 20 μM ISO stimulation, the macrophages in 5 μM and 20 μM group are in poor condition, so we set three concentrations including 0.1 μM, 0.5 μM, and 1 μM.TGF-β1 expression in macrophages was measured with RT-PCR.

### M1/M2 macrophage identification by flow cytometry

To identify macrophage M1/M2 populations, flow cytometry was performed. After blocked Fc receptors of heart cells removed from Sprague-Dawley rats with purified mouse anti-rat CD32 (2.0 μg: 10^6^ cells in 100 μl volume, BD, 550270), F4/80:APC (bio-rad, MCA497APCT), CD86: PE (Biolegend, 200,307), Arg1 (Novus, NBP1–32731) and FITC IgG (bio-rad, 1608) which is to identify Arg1 were used to mark M1 and M2 subpopulations. F4/80+/CD86+ cells were considered to be M1 macrophage, whereas F4/80+/Arg1+ were identified as M2 macrophages. Cells were analyzed using FCS Express V6.

### Co-culture of cardiac fibroblasts and macrophages

Briefly, hearts were removed from Sprague-Dawley rats aged 1–2 days under aseptic conditions and were placed in Dulbecco’s modified Eagle’s medium (DMEM)/F12 medium (Gibco; Gaithersburg, MD, USA). After washing with the DMEM/F12 medium, the atria and aorta were discarded. The ventricles were then minced with scissors into fragments < 1 mm^3^ and enzymatically digested for five 15 min cycles with 8 mL of D-Hanks containing 0.125% trypsin (Gibco). After centrifugation, the sediment was resuspended in DMEM/F12 medium supplemented with 15% FBS (HyClone; Logan, UT, USA). The fibroblast content of the cell suspension was removed and seeded by a differential attachment technique.

5 × 10^6^ cardiac fibroblasts were co-cultured with 1 × 10^6^ macrophages. The cells were treated with 20 μM ISO with or without BBR (1 μM) for 24 h, then the RNA was extracted from cells, and mRNA of α-SMA, collagen Iα, and collagen IIIα expression were detected by RT-PCR.. In our study, we tested the TGF β1 mRNA expression in macrophages after treatment with 10 μM and 20 μM ISO, and choose the 20 μM for the following experiments. The experiments were performed in 3 replicate wells. The experiments were repeated independently for 3 times.

### Proliferation assay

Primary peritoneal macrophages (2 × 10^6^ cells/well) of SD rats were cultured in 6-well plates as mentioned before. 1 μM BBR were incubated with ISO (20 μM)-induced macrophages for 24 h. The cardiac fibroblasts were cultured in 96-well plates, after serum starvation for 24 h, the cardiac fibroblasts were treated with supernatant from the macrophages (Four groups: CON, BBR 1 μM, ISO 20 μM, ISO 20 μM+ BBR 1 μM) for 24 h. Then, Cell Counting Kit-8 (Dojindo, CK-04) was used to analyze the proliferation of cardiac fibroblasts according to the manufacturer’s instructions. The optical density was detected at an absorbance of 450 nm using a Synergy HT microplate reader (Bio-Tek Instruments, Inc., Winooski, VT, USA). The cell proliferation state was expressed as the percentage cell proliferation compared with the control group, which was set at 100%. Six wells for each group. The experiments were repeated independently for 3 times.

### Co-culture of cardiomyocyte and macrophages

Briefly, hearts were removed from Sprague-Dawley rats aged 1–2 days under aseptic conditions and were placed in Dulbecco’s modified Eagle’s medium (DMEM)/F12 medium (Gibco; Gaithersburg, MD, USA). The fibroblast content of the cell suspension was removed by a differential attachment technique. After 48 h, the cardiomyocytes were co-cultured with macrophages. The cells were treated with 20 μM ISO with or without BBR (1 μM) for 24 h, then the RNA was extracted from cells, and mRNA of TGFβ, CTGF, and ANP expression were detected by RT-PCR. The experiments were performed in 3 replicate wells. The experiments were repeated independently for 3 times.

### Statistical analysis

Data were expressed as the mean ± SEM. Statistical analysis was performed using SPSS 13.0 (SPSS Inc.; Chicago, IL, USA) software. Data were analyzed by one-way ANOVA followed by Tukey’s post-hoc test. *P* < 0.05 was considered as statistically significant.

## Results

### Effect of berberine on ISO-induced cardiac fibrosis

**Fig. 1:**
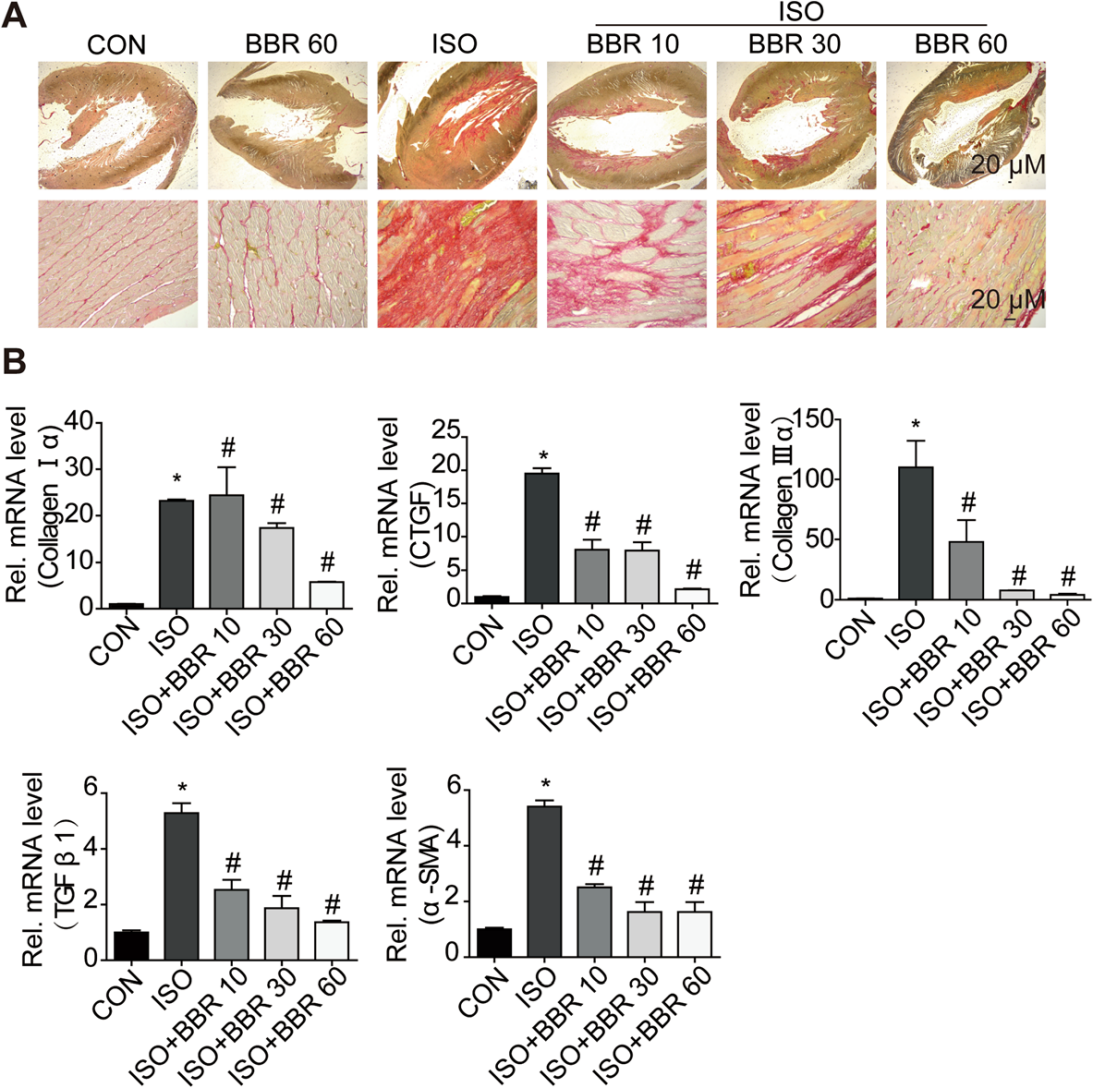
Effects of berberine on isoprenaline-induced cardiac fibrosis. The effect of three different daily doses of berberine (10 mg/kg/d, 30 mg/kg/d, and 60 mg/kg/d, respectively) on isoprenaline (ISO)-induced cardiac fibrosis, cardiac structural changes, and cardiac dysfunction. (**a**) On day 10 after ISO injection, rat heart sections were stained with picrosirius red. Magnification X10. (*n* = 6 rats per experimental group) (**b**) The expression of collagen Iα, collagen IIIα, connective tissue growth factor, transforming growth factor-β1, and α-smooth muscle actin was determined by reverse transcription polymerase chain reaction. (*n* = 6 per experimental group)

There are no significant differences of BW among the groups at the beginning of the experiment (Additional file 1: Table S1). The ratios of lung weight to body weight (LW/BW) of the ISO group and ISO + BBR group were not significantly different compared to that of the control group (Table 1). Furthermore, the ISO treated rats showed higher heart weight to body weight ratios (HW/BW) compared to that seen in the control rats; this increase was ameliorated by pretreatment with BBR (Table 1). Images of the heart and histological assessment showed that pretreatment with BBR ameliorated cardiac fibrosis in ISO administered rats (Fig. 1a). We also analyzed the expression patterns of collagenIα, collagen IIIα, CTGF, TGF-β1 and α-SMA, the key components in the process of cardiac fibrosis. Berberine (60 mg/kg) alone did not change the mRNA expression mentioned above in rat hearts (Additional file 2: Figure S1A). However, pretreatment with BBR yielded a pronounced reduction in the expression of these fibrotic markers after ISO induction (Fig. 1b).

**Table 1:** HW/BW ratio and LW/BW ratio in the indicated groups

Group	HW/BW (mg/g)	LW/BW (mg/g)
CON	3.02 ± 0.07	3.72 ± 0.19
ISO	4.26 ± 0.15^*^	4.22 ± 0.34
ISO + BBR 10	4.19 ± 0.09	3.96 ± 0.07
ISO + BBR 30	3.81 ± 0.13^#^	3.75 ± 0.12
ISO + BBR 60	3.74 ± 0.07^#^	3.42 ± 0.11

*p < 0.05 as compared with the CON group. #*p* < 0.05 vs ISO group

Abbreviations: *CON* the control group, *ISO* isoprenaline, *BBR* berberine, *LW/BW* lung weight to body weight, *HW/BW* heart weight to body weight

### Effect of berberine on cardiac structure and function after ISO treatment

**Fig. 2:**
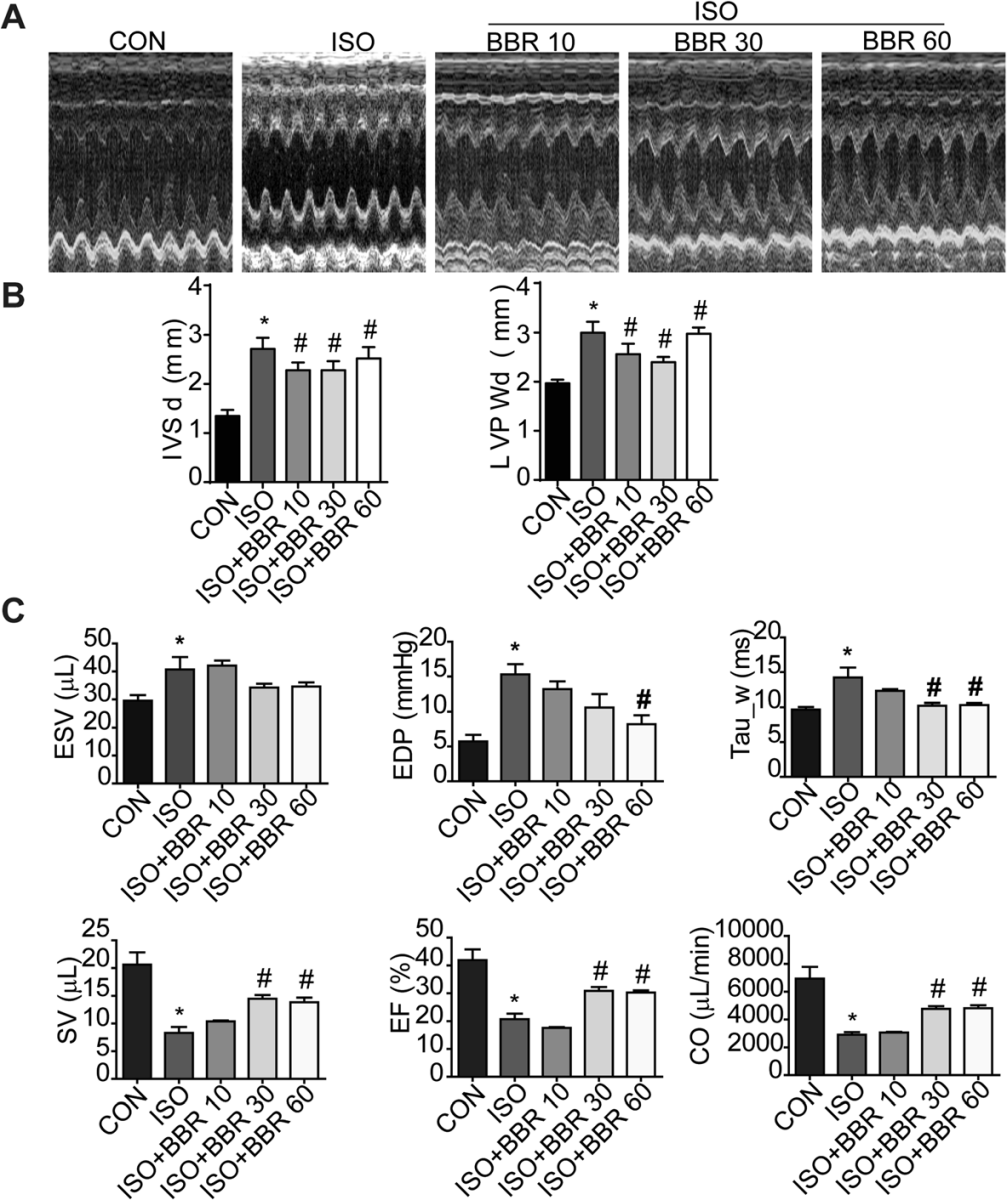
Effects of berberine on isoprenaline-induced cardiac dysfunction. (**a**) Representative M-mode images of the rat hearts. (**b**) Berberine (BBR) pretreatment attenuated an isoprenaline (ISO)-induced increase in the interventricular septum thickness at diastole and left ventricular end-diastolic posterior wall thickness. (*n* = 5–7 rats per experimental group) (**c**) Normalization of hemodynamic parameters with BBR pretreatment. (n = 5–6 rats per experimental group) *P < 0.05 as compared with the control group. #*p* < 0.05 vs. the ISO group. Abbreviations: CON, control group; ISO, isoprenaline; BBR, berberine; CTGF, connective tissue growth factor; TGF-β1, transforming growth factor β1; LVPWd, left ventricular end-diastolic posterior wall thickness; IVSd, interventricular septum thickness at diastole; EDP, end-diastolic pressure; ESV, end-systolic volume; Tau_w, time constant of isovolumic pressure decay; SV, stroke volume; EF, ejection fraction; CO, cardiac output

After 10 days of ISO injection, the rats showed increased IVSd and LVPWd. Berberine (60 mg/kg) alone did not affect the IVSd and LVPWd of rats (Additional file 2: Figure S1B and S1C). Berberine administration prevented these cardiac structural changes in ISO-treated rats as shown by the IVSd and LVPWd values in the ISO + BBR groups (Fig. 2 a and b). Figure 2c shows the results of the in vivo tests of cardiac function. Rats with sustained ISO stimulation showed reduced contractility as shown by a decreased SV, EF, and CO, and a deterioration in relaxation as indicated by an increased Tau_w. Rats pretreated with BBR demonstrated increased contractility and relaxation (Fig. 2c).

### Berberine inhibited macrophages infiltration and inflammatory factors expression in ISO-induced rat heart

**Fig. 3:**
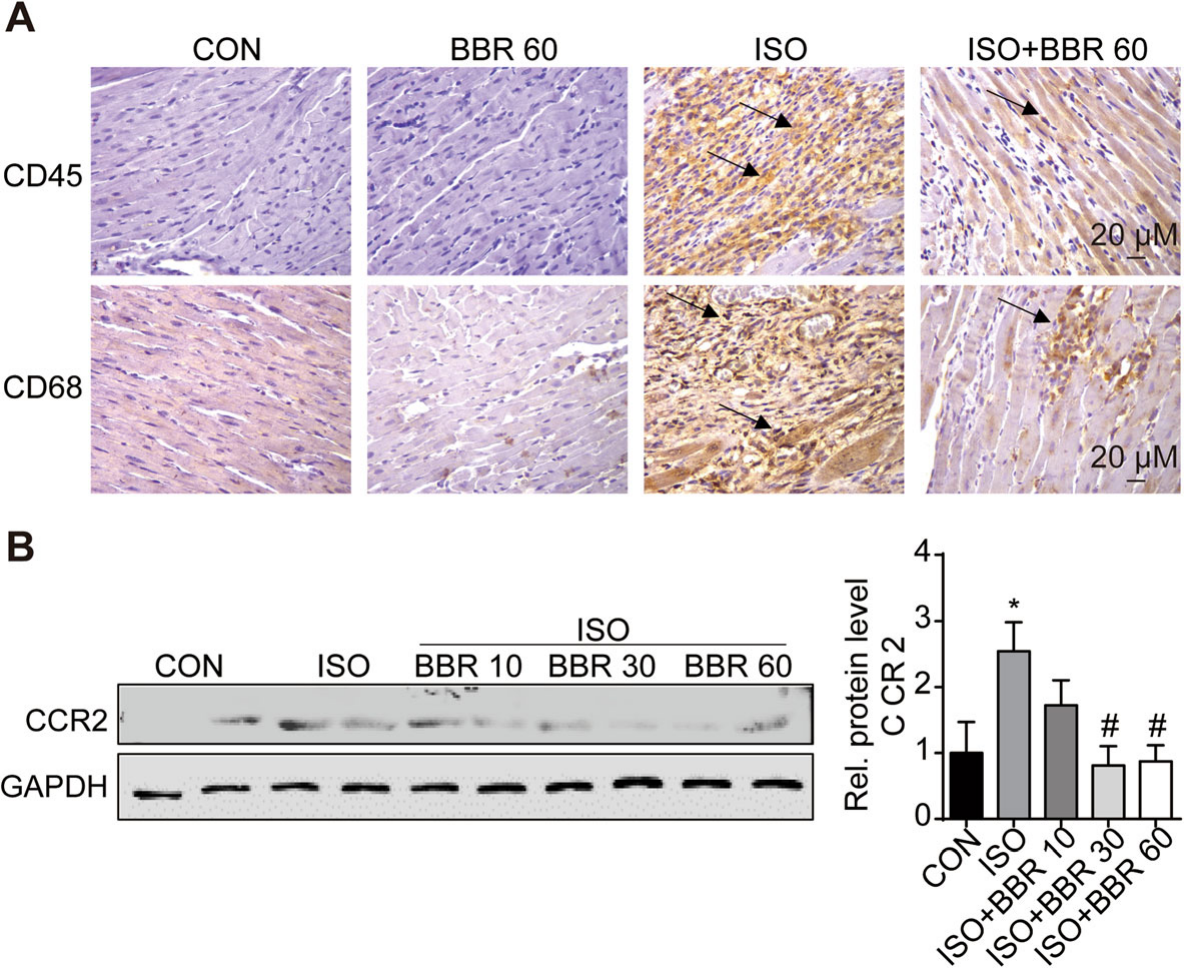
Effects of berberine on the infiltration of macrophages into the myocardium. (**a**) Macrophage infiltration in the rat hearts. Magnification × 400. (**b**) Representative blots and the quantitative results of C-C chemokine receptor 2. *p < 0.05 as compared with the control group. #p < 0.05 vs. the isoprenaline group. (n = 6 rats per experimental group)

**Fig. 4:**
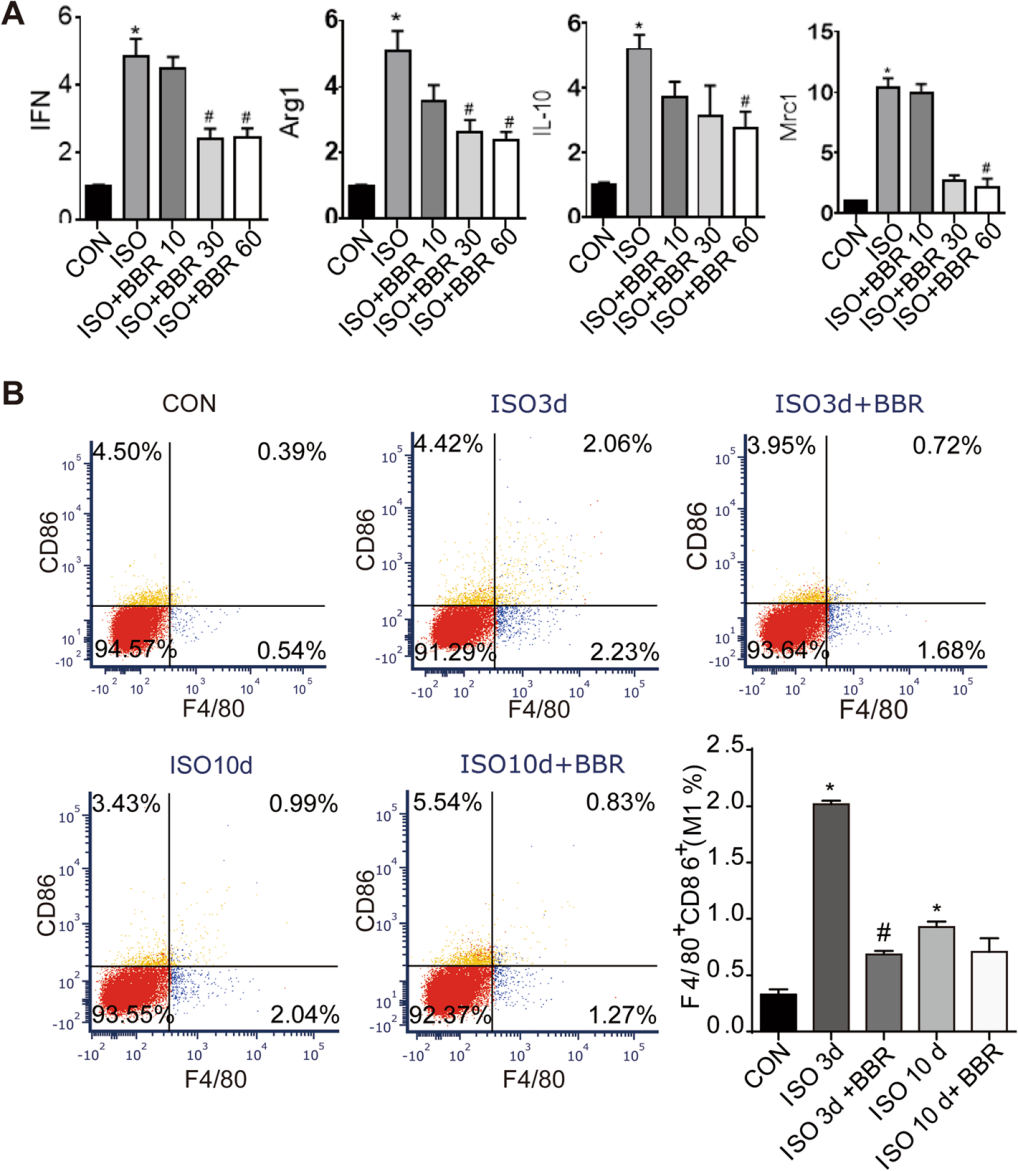
Effects of berberine on the M1 population of macrophages. (**a**) Quantitative analysis of mRNA expression of M1 marker, IFNγ and M2 markers, Arg1, IL-10 and Mrc1. (**b**) Representative dot plot of M1 subpopulations in control group and the rats injected with ISO 3 and 7 days later. M1 cells were labelled with F4/80 and CD86. *P < 0.05 as compared with the control group. #P < 0.05 vs. the corresponding ISO group

**Fig. 5:**
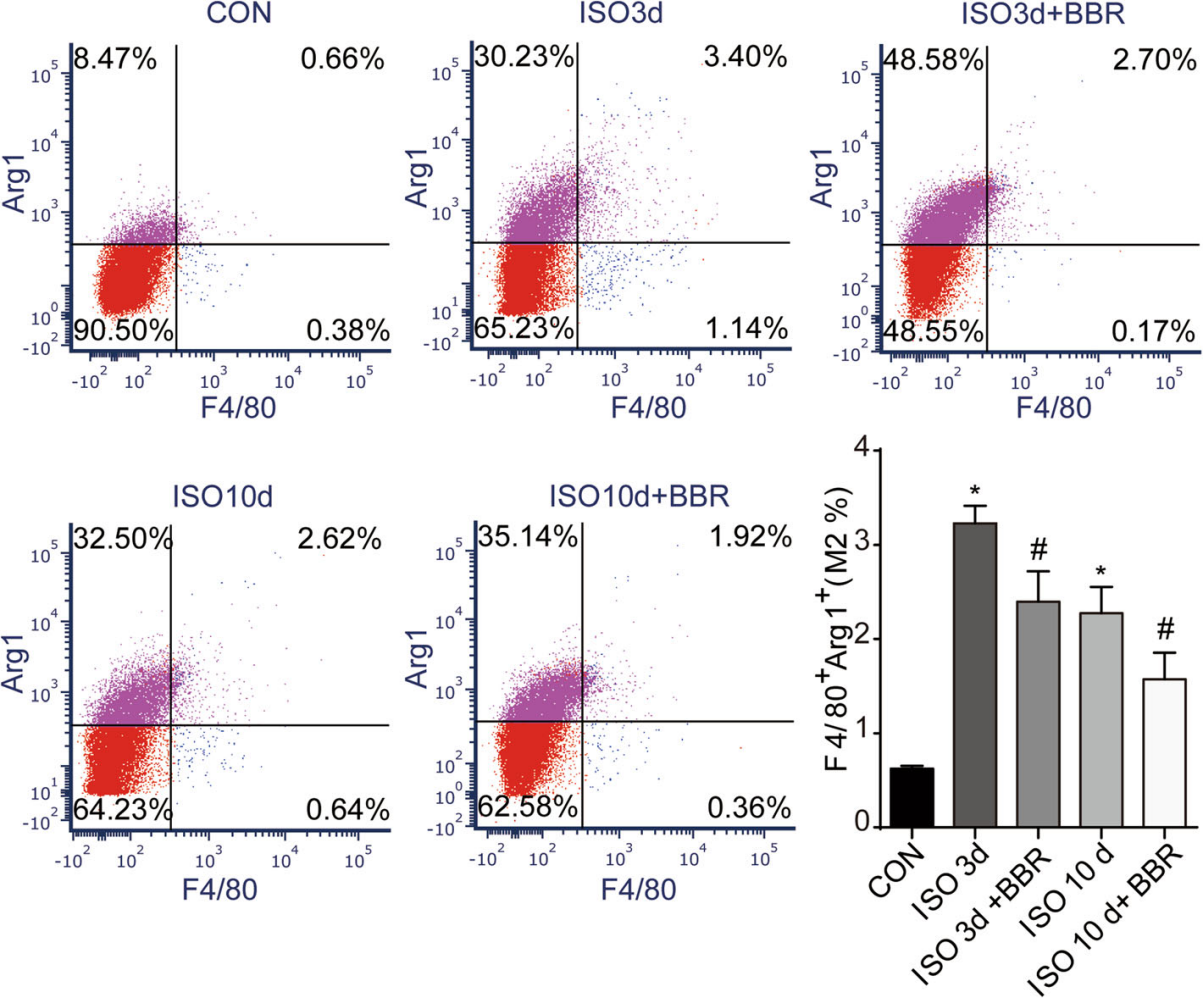
Effects of berberine on the M2 population of macrophages. Representative dot plot of M2 subpopulations in control group and the rats injected with ISO 3 and 7 days later. M2 cells were labelled with F4/80 and Arg1. *P < 0.05 as compared with the control group. #P < 0.05 vs. the corresponding ISO group

As macrophages are activated early in the early stage of heart injury and always been found in close proximity to collagen-producing myofibroblasts, we tested the infiltration of macrophages by immunolabeling straining, RT-PCR and Western blot. Results showed that compared with hearts of the rats in the ISO group, rats pretreated with BBR exhibited signs of a blunted macrophage infiltration response, as indicated by a reduction in the number of cells immunolabeling with CD45 and CD68 (Fig. 3a). In line with the immunohistochemical staining, western blot analysis showed lower levels of CCR2 proteins in the hearts from rats assigned to the three different BBR pretreatment dosages (Fig. 3b). Besides, to further analyse M1 and M2 polarization, RT-PCR and flow cytometry assay was performed. The results of RT-PCR showed that BBR reduced mRNA expression of the M1 and M2 markers (Fig. 4a). As Fig. 5b shows, the M1 fraction labelled by CD86 was reduced by BBR at 3 and 7 days after ISO injection, especially at 3 days, BBR induced an approximately 3-fold decrease in M1’s infiltration in myocardium. As for M2 macrophages, BBR also decreased their level at 3 and 7 days after ISO injection although the impact is smaller compared to M1 (Fig. 5).

### Berberine reduced TGF-β1/smads signaling pathway in ISO-induced rat heart

**Fig. 6:**
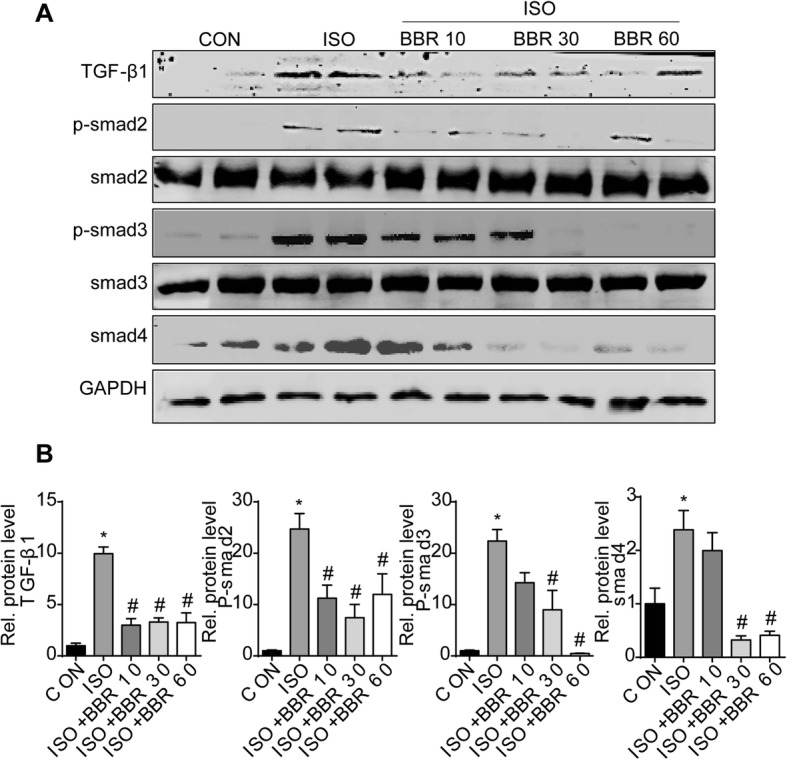
Effects of berberine on the transforming growth factor-β1/smads pathway. (**a**) Representative blots of transforming growth factor-β1, p-smad2, smad2, p-smad3, smad3, and smad4 in the heart tissues of rats in the indicated groups. (**b**) Quantitative results. (n = 6 rats per experimental group)

Then we investigated whether signal transduction via the TGF-β1/smads signaling pathway was relevant to the actions of BBR in ISO-induced cardiac fibrosis. Results showed that the TGF-β1/smads signaling pathway was activated in ISO-induced rat hearts, and this activation was blocked in the hearts of rats pretreated with BBR (Fig. 6 a and b).

**Fig. 7:**
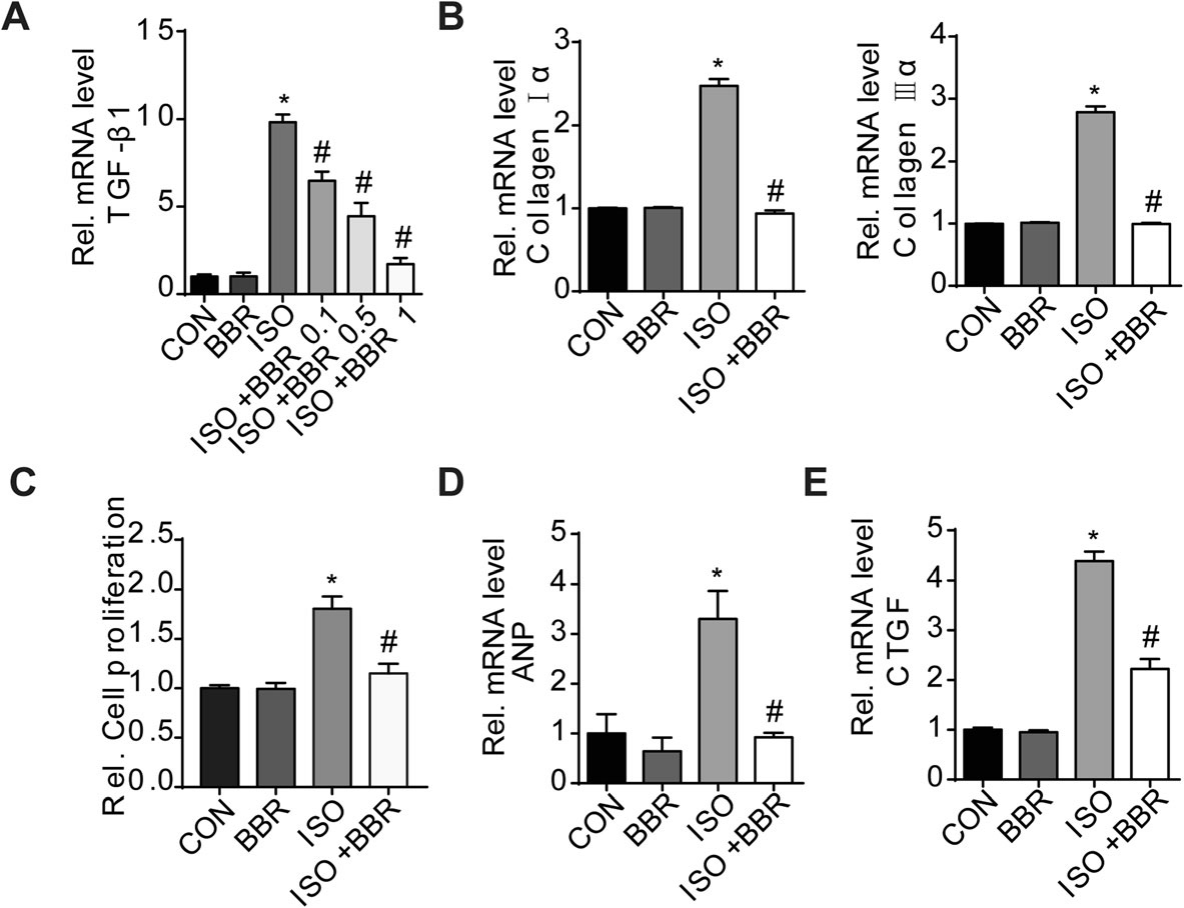
Effects of berberine on the production of transforming growth factor-β1 in macrophages, and on cardiac fibroblasts and cardiomyocytes which was co-cultured with macrophages. To investigate transforming growth factor (TGF)-β1 production in macrophages, different doses of berberine (0.1 μM, 0.5 μM, and 1 μM) were incubated with isoprenaline (ISO)-induced macrophages. The interaction between macrophages and fibroblasts or cardiomyocytes under ISO stimulation was evaluated, by macrophage/fibroblast or macrophage/cardiomyocyte co-culture. (**a**) TGF-β1 expression in macrophages was evaluated by reverse transcription polymerase chain reaction (RT-PCR). (**b**) The expression of markers of fibrosis (collagen Iα, and collagen IIIα) in fibroblasts induced by ISO-stimulated macrophages was determined by RT-PCR. (**c**) Cardiac fibroblast proliferation state in the indicated groups. (**d** and **e**) The expression of ANP and CTGF in cardiomyocytes co-cultured with macrophages in the indicated groups. *P < 0.05 as compared with the control group. #P < 0.05 vs. the ISO group

To further determine whether the action of BBR in the modulation of the TGF-β1/smads signaling pathway was associated with macrophages directly, we examined the effect of BBR on TGF-β1 production from macrophages in response to ISO stimulation. As shown in Fig. 7a, ISO induced an approximately 10-fold increase in TGF-β1 production. However, BBR treatment resulted in a concentration-dependent inhibition of TGF-β1 production. Next, we investigated whether macrophages exposed to ISO affect the expression of fibrotic markers in fibroblasts. We co-cultured macrophages and fibroblasts, and found that fibrotic marker expression (collagen Iα, and collagen IIIα) increased in fibroblasts co-cultured with ISO-stimulated macrophages. BBR treatment of ISO-stimulated macrophages decreased the expression of fibrotic markers in fibroblasts (Fig. 7b).

Cardiac fibroblasts proliferation is an important factor in cardiac fibrosis. To clarify whether macrophages exposed to ISO affect cardiac fibroblast proliferation, and whether BBR treatment has an effect on it, we cultured cardiac fibroblasts with supernatant from the macrophages (Four groups: CON, BBR 1 μM, ISO 20 μM, ISO 20 μM+ BBR 1 μM) respectively. It was found that BBR treatment inhibited cardiac fibroblasts proliferation at this situation (Fig. 7c).

β-AR stimulation induces synthesis and secretion of growth factors in cardiac myocytes that affect on cardiac fibroblast activation [18]. As shown in Fig. 7 d and e, when co-cultured with macrophages, ANP and CTGF expression in cardiomyocytes increased significantly after ISO stimulation, moreover, BBR treatment reversed the increase. It indicate that cardiomyocytes may also play a role in this situation, but the specific mechanism need to be investigated further.

## Discussion

Our findings demonstrate that 2 weeks of pretreatment with BBR prevented LV fibrosis and dysfunction caused by a daily administration of ISO. These effects were accompanied by a reduction in the expression of fibrotic markers and macrophage infiltration. Furthermore, we found that the TGF-β1/smads signaling pathway induction and CCR2 expression after ISO treatment were inhibited by BBR. In vitro studies verified that BBR treatment resulted in a concentration-dependent inhibition of TGF-β1 production in macrophages. In order to provide a mechanistic link between macrophages and fibroblasts, we studied an in vitro co-culture system in which macrophages were incubated with fibroblasts. ISO-stimulated macrophages were able to stimulate fibroblasts to produce fibrotic markers, and BBR inhibited the production of fibrotic markers by fibroblasts co-cultured with ISO-stimulated macrophages.

Cardiac fibrosis is characterized by the over deposition of myocardial interstitial collagen and altered cardiac function. Low doses of ISO (0.3 to 6 mg/kg) have been used to induce cardiac hypertrophy and widespread cardiac fibrosis. ISO administration causes severe stress in the myocardium due to the activation of the adrenergic system and is associated with the activation of transduction mechanisms and an increased expression of fibrotic factors, leading to cardiac remodeling and dysfunction [19]. In this study, we induced cardiac fibrosis in the rat model using subcutaneously injected-ISO (5 mg/kg).

Antifibrotic therapy would be beneficial for the treatment of patients with heart failure. Previous studies have focused on the effect of BBR in various models of heart injury, including a high-fat diet and strephtozotocin induced-type 2 diabetes model [20], a pressure overload-induced cardiac hypertrophy model [21], a left anterior descending coronary artery ligation model [22], a porcine cardiac myocin-induced experimental autoimmune myocarditis model [23], and a high-dose ISO (85 mg/kg) injection-induced heart injury model [9]. In these models, BBR has demonstrated an important cardioprotective effect. However, the mechanisms underlying the cardioprotective effect of BBR remain unclear. In this study, low-dose ISO injection-induced cardiac fibrosis model was investigated, and a new mechanism was suggested during the treatment period using BBR in the process of cardiac fibrosis.

It has long been known that macrophages are nearly always found close to collagen-producing myofibroblasts; however, the role of macrophages in mediating the fibrotic response is complex. Depending on CCR2 signaling, Ly-6C^high^ monocytes produced in the bone marrow are released into the blood and subsequently travel to the tissues where they participate in the host’s initial immune response [24, 25]. After heart injury, M1 macrophages are mainly proinflammatory, and M2 macrophages are mainly reparative. The transition from M1 to M2 macrophages after heart injury may be beneficial for heart repair, but uncontrolled or prolonged activation of M2 macrophages may eventually contribute to extensive cardiac fibrosis by triggering the accumulation of the extracellular matrix. Westermann et al. have shown that TGFβ-producing inflammatory cells contribute to diastolic dysfunction in human heart failure. Furthermore, M2 macrophages are a prominent source of TGF-β, which is one of the most important cytokines that promotes the differentiation of fibroblasts into collagen-producing myofibroblasts. Studies have also connected M2 macrophage-released TGF-β with vessel fibrosis in hypertension. In this study, we found that the effect of BBR were closely associated with its regulation of macrophages. On one side, BBR induced M1 macrophages decrease in the heart after ISO treatment, on the other side, BBR also reduces M2 macrophages after ISO treatment. This suggests that BBR may inhibit the infiltration of macrophages in the ISO-treated hearts, leading to protection of the heart.

TGF-β has been identified as a key regulator of cardiac fibrosis. Phosphorylation of Smad2 and Smad3 that forms a complex with Smad4 moves into nucleus to regulate downstream proteins [26], leading to collagen synthesis. TGF-β could promote the transformation and proliferation of myocardial fibroblasts through the induced Smads proteins [27]. BBR could down-regulated the expression of TGF-β/Smads proteins caused by ISO in the heart tissue. It suggested that BBR might intervene with the myocardial fibrosis process through regulating TGF-β/Smads signal transduction pathways.

The current study has several limitations. Firstly, we didn’t investigate the influence of BBR on other cells induced by ISO. In addition, the proportion of macrophage’s contribution in cardiac fibrosis have not been measured. Further investigations are required to elucidate the specific mechanisms of macrophage effects on ISO-induced cardiac remodeling.

## Conclusions

In conclusion, this study demonstrates the cardioprotective effect of BBR on ISO-induced cardiac fibrosis in rats. The mechanism of BBR may be via the inhibition of the infiltration of macrophages. We propose that BBR may offer a potentially effective approach to retard the process of cardiac injury caused by rapid developing stress conditions.

## Supplementary information

**Additional file 1: Table S1.** BW in the indicated groups at baseline indicating that there are no significant differences of BW among the groups at the beginning of the experiment.

**Additional file 2: Figure S1.** Indicating that BBR (60 mg/kg) showed no obvious effect in rat hearts. (A) The expression of collagen I α, collagen III α, connective tissue growth factor, transforming growth factor-β1, and α-smooth muscle actin was determined by reverse transcription polymerase chain reaction. (B) The interventricular septum thickness at diastole and left ventricular end-diastolic posterior wall thickness. (C) Representative M-mode images of the rat hearts. (D and E) Effects of BBR on the transforming growth factor-β1/smads pathway and CCR2 expression in rat hearts. (F) Quantitative analysis of mRNA expression of M1 marker, IFN and M2 markers, Arg1, and IL-10 in the indicated groups.

## Data Availability

The datasets used and/or analysed during the current study are available from the corresponding author on reasonable request.

## Abbreviations

BBR: Berberine
CCR2: C-C chemokine receptor 2
CO: Cardiac output
CTGF: Connective tissue growth factor
DAPI: 4′, 6-Diamidine-2′-phenylindole dihydrochloride
EDP: End-diastolic pressure
EF: Ejection fraction
ESV: End-systolic volume
GAPDH: Glyceraldehyde-3-phosphate dehydrogenase
HW/BW: Heart weight to body weight ratios
IL-1β: Interleukin-1β
ISO: Isoprenaline
IVSd: Interventricular septum thickness at diastole
LV: Left ventricle
LVPWd: Left ventricular posterior wall thickness
LW/BW: Lung weight to body weight
PSR: Picrosirius red
RT-PCR: Reverse transcription polymerase chain reaction
SV: Stroke volume
Tau_w: Time constant of isovolumic pressure decay
TGF-β1: Transforming growth factor-β1
TNFα: Tumor necrosis factor α
α-SMA: α-Smooth muscle actin
