# Supplementary material for: Protective role of berberine in isoprenaline-induced cardiac fibrosis in rats
Source: BMC Cardiovasc Disord. 2019 Oct 15;19:219. doi: 10.1186/s12872-019-1198-9 (PMC6792193; doi:10.1186/s12872-019-1198-9)
Supplement: Supplementary file 1 — Additional file 1: Table S1. BW in the indicated groups at baseline indicating that there are no significant differences of BW among the groups at the beginning of the experiment. [file 12872_2019_1198_MOESM1_ESM.docx]

Table S1. BW in the indicated groups at baseline

| Group | BW (g) |
| --- | --- |
| CON | 229.9 ± 2.95 |
| ISO | 221.3 ± 2.65 |
| ISO+BBR 10 | 220.9 ± 3.36 |
| ISO+BBR 30 | 219.6 ± 3.38 |
| ISO+BBR 60 | 220.2 ± 1.36 |
