# Supplementary figures and images for: Protective role of berberine in isoprenaline-induced cardiac fibrosis in rats
Source: BMC Cardiovasc Disord. 2019 Oct 15;19:219. doi: 10.1186/s12872-019-1198-9 (PMC6792193; doi:10.1186/s12872-019-1198-9)

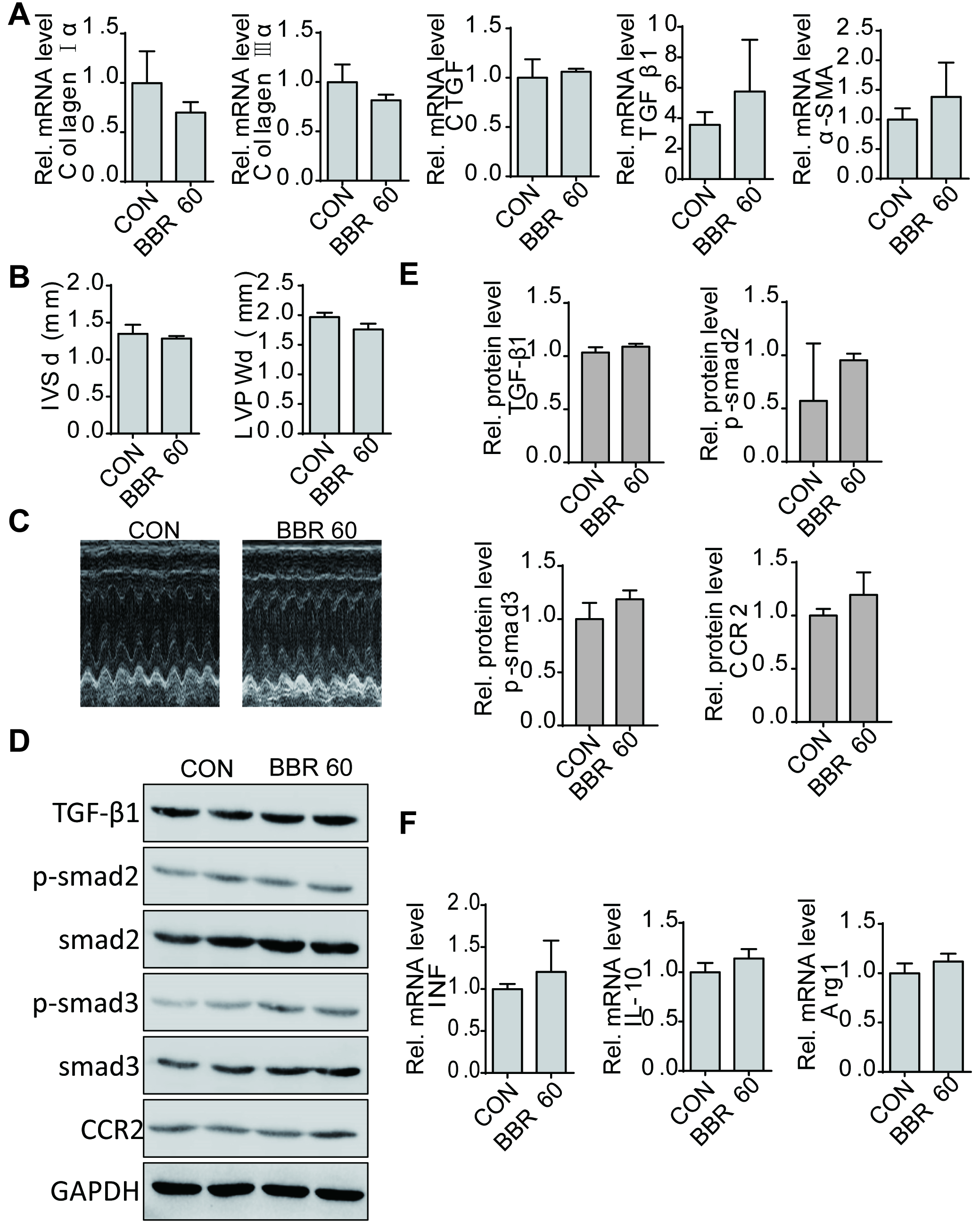

Supplement: Supplementary file 2 — Additional file 2: Figure S1. Indicating that BBR (60 mg/kg) showed no obvious effect in rat hearts. (A) The expression of collagen I α, collagen III α, connective tissue growth factor, transforming growth factor-β1, and α-smooth muscle actin was determined by reverse transcription polymerase chain reaction. (B) The interventricular septum thickness at diastole and left ventricular end-diastolic posterior wall thickness. (C) Representative M-mode images of the rat hearts. (D and E) Effects of BBR on the transforming growth factor-β1/smads pathway and CCR2 expression in rat hearts. (F) Quantitative analysis of mRNA expression of M1 marker, IFN and M2 markers, Arg1, and IL-10 in the indicated groups. [file 12872_2019_1198_MOESM2_ESM.tif]
